# Effects of external load magnitude and carriage techniques on dynamic stability and work performance during fatigued rescue sprint tasks in firefighters

**DOI:** 10.3389/fbioe.2025.1671642

**Published:** 2025-10-02

**Authors:** Enmeng Jiang, Yan Shi, Xinxin Zhang, Weiguo Liu

**Affiliations:** ^1^ School of Teacher Development, Shaanxi Normal University, Xi’an, China; ^2^ Department of Exercise Science, School of Physical Education, Shaanxi Normal University, Xi’an, China; ^3^ College of Physical Education and Health, Guangxi Normal University, Guilin, China

**Keywords:** firefighter, external load magnitude, carrying technique, dynamic stability, work performance

## Abstract

**Background:**

This study aims to investigate the effects of different external load magnitudes and carrying techniques on dynamic stability and work performance during fatigued rescue sprint tasks in firefighters, thereby providing theoretical and practical support for injury prevention and performance enhancement in firefighting rescues.

**Methods:**

A total of 126 professional healthy male firefighters performed 30-m rescue sprints under simulated fatigue in a 3 × 3 two-factor experimental design combining three external load magnitudes (10 kg, 20 kg, 30 kg) and three carrying techniques (shoulder-carry, bosom-carry, hand-carry).

**Results:**

External load magnitude had no significant effect on margin of stability in the medial and lateral directions (MoS_ml_) (P > 0.05), but the margin of stability in the anterior and posterior directions (MoS_ap_) was significantly lower under 10 kg load tasks compared to 20 and 30 kg loads (P < 0.05). The hand-carry technique significantly increased MoS_ap_ (P < 0.05) but significantly reduced MoS_ml_ (P < 0.05). For 20 kg loads, hand-carrying resulted in significantly increased MoS_ml_ compared to 10 and 30 kg tasks (P < 0.05), while bosom-carrying with 10 kg loads or shoulder-carrying with 20 kg loads significantly reduced MoS_ml_ (P < 0.05). Center of mass (CoM) work was significantly higher under 20 kg load tasks (P < 0.05). It was also significantly higher when using the hand-carry technique (P < 0.05), particularly when hand-carrying 20 kg loads (P < 0.05). Both external load magnitude and carrying technique significantly affected hip joint work (P < 0.05). Hip joint work was significantly higher under 10 kg loads (P < 0.05), and significantly greater when using hand-carrying compared to bosom-carrying (P < 0.05).

**Conclusion:**

It is recommended that firefighters choose carrying techniques based on specific load conditions: avoiding hand-carrying moderate loads, while shoulder carry for moderate to large loads, and bosom carry for small loads. Additionally, hip joint function training should be emphasised in daily routines to improve body control, reduce injury risk during rescue tasks, and enhance overall task performance.

## 1 Introduction

Due to the unique nature of their profession, firefighters’ ability to perform emergency rescue tasks plays a critical role in maintaining social order and public safety (Tang et al., 2025; Zhang et al., 2024). Compared to most occupations, firefighting is characterized by high frequency, elevated risk, and urgent time constraints (Tang et al., 2025). The high frequency and risk render firefighters particularly susceptible to fatigue, while the urgency of tasks often necessitates performing rescue tasks while sprinting (Hu et al., 2024). Fatigue is an essential physiological response in firefighting tasks due to prolonged exercise and high-intensity demands. In fact, during rescue runs performed under fatigue, maintaining good dynamic stability and achieving efficient work are critical to ensuring task completion and individual safety (Maxwell et al., 2024; Liao et al., 2024). However, these capacities are often constrained by the biomechanical demands and motor control challenges imposed by external load magnitude and carrying techniques (Tang et al., 2025; Winter, 2009). Therefore, examining how different load magnitudes and carrying techniques influence firefighters’ stability and efficiency is of significant theoretical and practical value, particularly under fatigue conditions.

With the advancement of exercise biomechanics and occupational health research, numerous studies have investigated firefighter movement under load-bearing or fatigued states (Chen et al., 2024; Gregory et al., 2008; Tang et al., 2025; Vaulerin et al., 2020; Wolkow et al., 2015; Zhang et al., 2024). Evidence shows that running with loads increases metabolic demand, alters gait, and raises joint stress (Tang et al., 2025; Zhang et al., 2024), while fatigue impairs muscular response, joint stability, and neuromuscular control (Gregory et al., 2008; Vaulerin et al., 2020). However, most prior studies have treated these variables in isolation, focusing only on load weight or carrying posture (Chen et al., 2024; Tang et al., 2025). Few have explored the combined effects of load magnitude and carrying technique, even though these two factors interact dynamically in real rescue contexts to determine performance outcomes. More importantly, most experimental designs remain limited to non-fatigued or walking scenarios (Maxwell et al., 2024; Zhang et al., 2024); there are limitations on the ecological validity of high fatigue and rapid running that characterize real-life rescue situations. Regarding dependent variables, although dynamic stability and work performance (mechanical work output) are critical indicators of firefighters’ task performance, current research often emphasizes static stability frameworks (Aljaroudi et al., 2023; Hof et al., 2005; Singh et al., 2019) or gear optimization (Tang et al., 2025; Zhang et al., 2024), while overlooking dynamic stability during high-speed tasks and the biomechanical mechanisms of energy expenditure. Addressing these gaps requires a more comprehensive evaluation of how load magnitude and carrying technique jointly influence firefighter biomechanics under fatigue.

In summary, this study adopts a 3 (external load magnitude: large load 30 kg, moderate load 20 kg, small load 10 kg) × 3 (carrying technique: shoulder-carry, bosom-carry, hand-carry) two-factor experimental design to examine their combined effects on firefighters’ dynamic stability and work performance during fatigued rescue sprints. The findings provide theoretical foundations and practical guidance for injury prevention and performance enhancement in real-world firefighting scenarios.

## 2 Materials and methods

### 2.1 Participants

The required sample size was calculated using G*Power software, with statistical testing based on two-way ANOVA. Referring to Zhang et al. (2024), the effect size f was set at 0.4, the Type I error rate ɑ at 0.05, and statistical power (1-β) at 0.85. Considering a 10% dropout rate, the final minimum sample size was 113 participants. Therefore, 126 professional healthy male firefighters who met the inclusion criteria were randomly selected as participants. Using a random number table, they were assigned to one of nine groups defined by external load magnitude and carrying technique combinations. Basic characteristics of participants are presented in Table 1. The inclusion criteria were based on Zhang et al. (2024) and included 1. All participants used a heel strike running technique (self-reported); 2. All participants were right-leg dominant, as determined by a four-trial ball kicking test in which the leg used most frequently was identified as dominant; 3. No history of injury or medication use within the past year. All participants signed an informed consent form before participation. This study was conducted following the Declaration of Helsinki and was approved by the Ethics Committee of Guangxi Normal University.

**TABLE 1:** Basic characteristics of participants.

Group	N	Height (cm)	Weight (kg)	Age (years)	Years of Firefighting service
10 kg × Shoulder-carry	14	171.81 (4.86)	67.56 (10.19)	24.63 (2.87)	4.25 (2.38)
10 kg × Bosom-carry	14	175.73 (6.89)	76.19 (8.21)	24.63 (2.12)	3.63 (1.80)
10 kg × Hand-carry	14	172.64 (7.46)	69.70 (7.84)	25.29 (4.20)	3.29 (2.05)
20 kg × Shoulder-carry	14	174.21 (5.37)	68.96 (8.27)	24.84 (3.26)	4.17 (2.55)
20 kg × Bosom-carry	14	173.56 (3.98)	72.85 (9.03)	25.33 (3.50)	3.78 (1.76)
20 kg × Hand-carry	14	172.59 (6.28)	70.55 (7.95)	23.59 (2.89)	3.56 (2.09)
30 kg × Shoulder-carry	14	171.99 (5.34)	71.69 (8.36)	25.63 (3.27)	4.09 (2.57)
30 kg × Bosom-carry	14	174.36 (6.15)	75.98 (10.03)	23.37 (2.39)	3.93 (1.56)
30 kg × Hand-carry	14	174.82 (7.32)	76.21 (9.79)	24.51 (4.11)	3.86 (2.01)

All data are presented as mean (standard deviation); the same applies below.

### 2.2 Experimental design and materials

**FIGURE 1:**
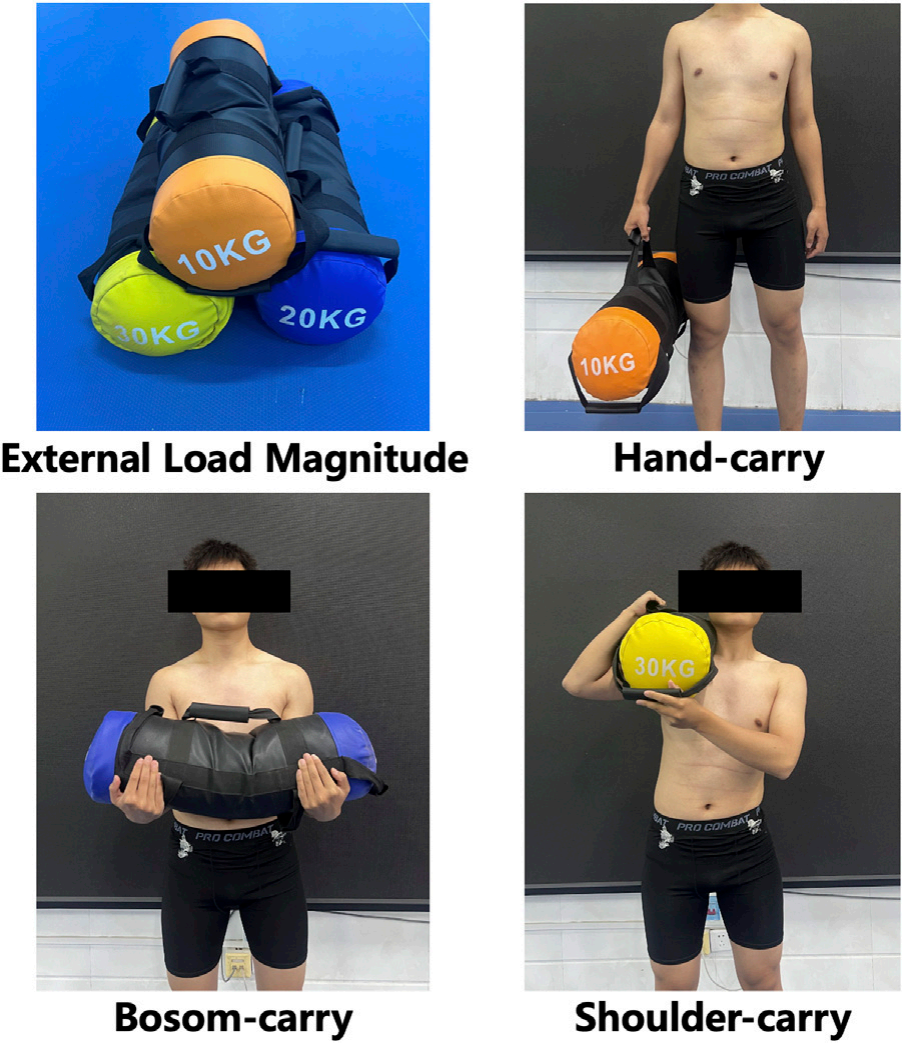
Schematic diagram of external load magnitude and carrying technology.

The experimental test rescue project involved firefighters performing a 30-m rescue sprint while carrying a specified weight using designated carrying techniques in a fatigued state. A 3 × 3 two-factor experimental design was employed. The first factor, external load magnitude, was determined based on typical firefighting operational or training scenarios, and included three commonly encountered load conditions: small load (10 kg, approximating the weight of a fire hose), moderate load (20 kg, approximating the weight of a gas can), and large load (30 kg, approximating the weight of a fire ladder). The second factor, carrying technique, was selected according to frequently used methods in firefighting and included shoulder carry, hand carry, and bosom carry (as illustrated in Figure 1). The loads were simulated using flexible multifunctional fitness sandbags (KR Fitness), replicating realistic rescue weights during the sprint tasks.

### 2.3 Experimental equipment

A treadmill (Merrick Phantom X1, China) and a Polar heart rate monitor (H9, Finland) were used to induce fatigue in participants. Kinematic data was collected using eight infrared motion capture cameras (Oqus 600+, Qualisys AB, Sweden) operating at 200 Hz, while kinetic data was obtained via two 3D force platforms (Kistler and AMTI, 2000 Hz). All systems were synchronized via an analog-to-digital converter to ensure temporal alignment.

### 2.4 Testing procedures and methods

#### 2.4.1 Fatigue model construction

Before testing, participants wore compression tights and performed a standardized 10-min warm-up, which included 5 min of light jogging followed by 5 min of stretching, primarily targeting the hamstrings and quadriceps. After the warm-up, firefighters wore heart rate monitors to undergo fatigue induction. The fatigue protocol was designed to simulate the typical running conditions encountered during firefighting rescue tasks. It was constructed based on the treadmill training model developed by Gordon et al. (2010) and further adapted by Xia et al. (2017). Specifically, the treadmill protocol began with an initial 5 km/h speed and a 0% incline. The speed remained constant for the first 14 min while the incline increased by 2% every 2 min until reaching 14%. Thereafter, the incline remained fixed, and the running speed was increased by 0.5 km/h every minute. Following previous studies (Xia et al., 2017; Zhang et al., 2024), participants were considered to have reached a fatigued state once both of the following criteria were met: (1) heart rate reached 90% of the individual’s age-predicted maximum heart rate, and (2) the participant could no longer maintain the required running speed.

#### 2.4.2 Static and dynamic data collection

**FIGURE 2:**
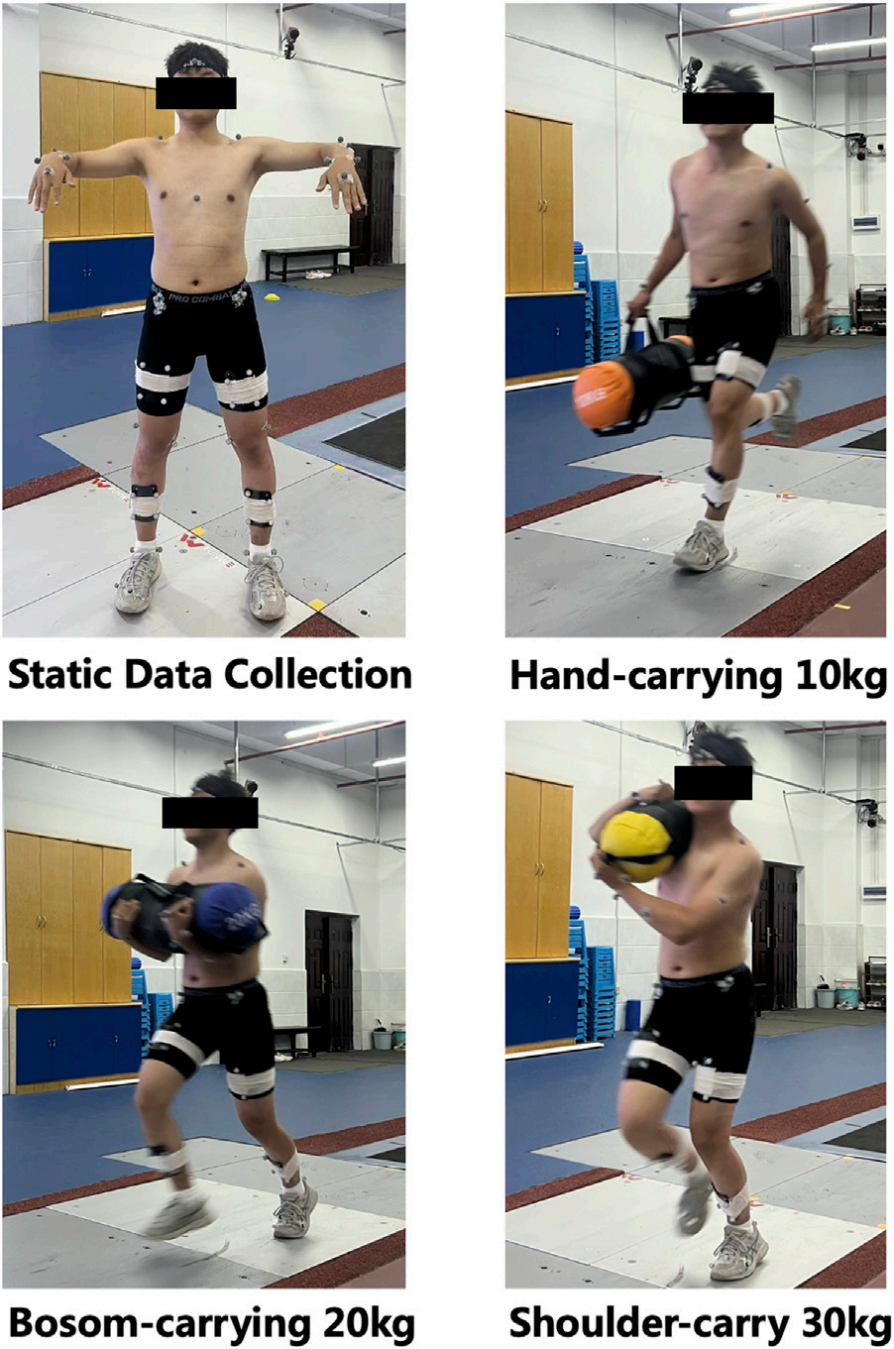
Data acquisition process diagram.

Upon reaching the fatigue state, static lower limb data were collected from all participants for subsequent modeling and analysis. Participants were instructed to stand in the center of the testing area with their feet shoulder-width apart and their eyes looking forward (see Figure 2). Following static capture, dynamic data collection commenced. During dynamic trials, participants performed a 30-m rescue sprint at maximum running speed while carrying the assigned load using the designated carrying technique (see Figure 2). Each participant underwent three tests and collected valid data. The final results were presented as the average of the three valid trials to reduce random variation. A trial was considered valid if the participant’s foot remained entirely within the force plate boundaries and no reflective marker was lost during the data collection (Zhang et al., 2024).

### 2.5 Experimental parameters and data processing

#### 2.5.1 Phase definition

The stance phase during running is critical for buffering load impact and generating push-off force. It is also a phase with a high risk of sports injuries, especially when the body is fatigued, increasing the injury incidence (Winter, 2009; Zhang et al., 2024). Therefore, this study selected the onset of the stance phase (the instant the dominant leg’s heel contacts the force plate) and the entire stance phase to analyze dynamic stability and work performance, respectively. Heel contact was defined as the moment when the vertical ground reaction force (vGRF) > 10 N, and the entire stance phase was determined from heel contact until the toe left the force plate (vGRF < 10 N) (Zhang et al., 2024).

#### 2.5.2 Data processing for dynamic stability parameters

Based on previous research, the Margin of Stability (MoS) was introduced to evaluate body dynamic stability (Hof et al., 2005). Three-dimensional kinematic and kinetic data were processed using the biomechanics analysis software Visual 3D (C-Motion Inc.), with a fourth-order Butterworth low-pass digital filter applied at cut-off frequencies of 14 Hz and 50 Hz, respectively (Koblbauer et al., 2014; Winter, 2009). The calculation formulas for MoS are as follows:

$$\text{CM}=\text{pCoM}+\frac{\text{vCoM}}{\sqrt{g/l}}$$

MoS=Bmax−CM

**FIGURE 3:**
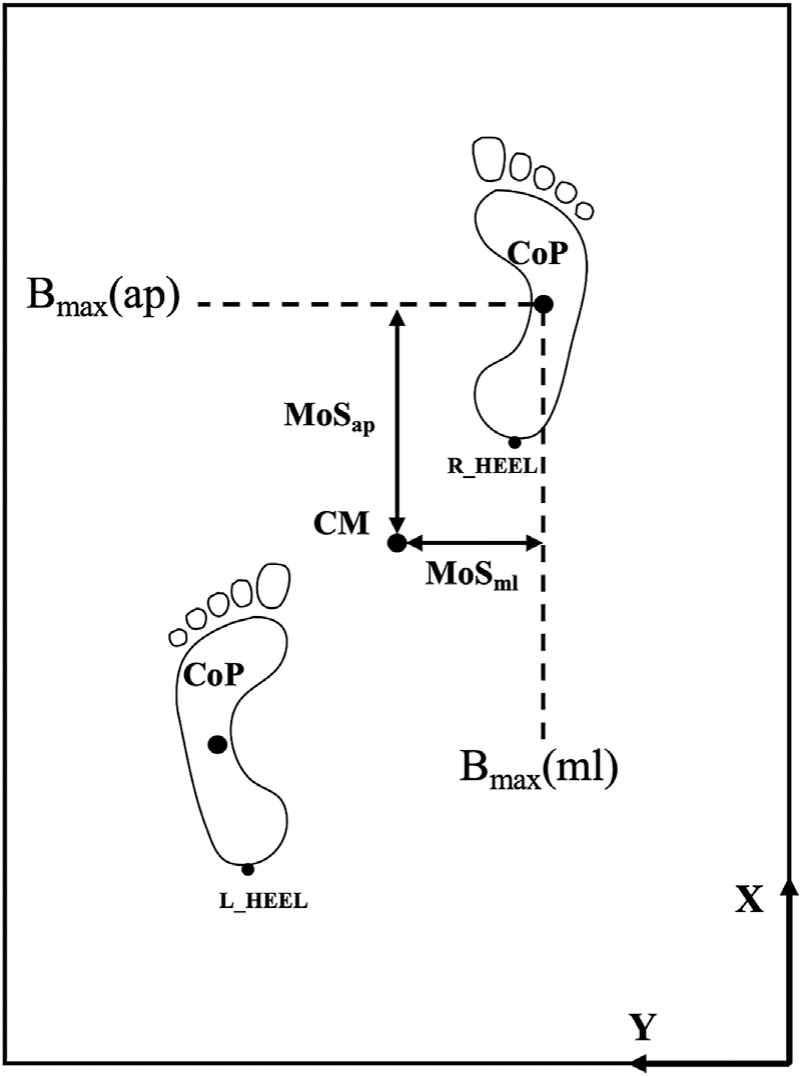
Schematic diagram of the margin of stability. CM refers to the extrapolated center of mass; B_max (ap) and B_max (ml) represent the furthest boundaries of the base of support in the anterior-posterior and medial-lateral directions, respectively; CoP stands for the center of pressure; MoS_ap_ and MoS_ml_ denote the Margin of Stability in the anterior-posterior and medial-lateral directions, respectively; R_HEEL and L_HEEL refer to the right and left heel, respectively.

In the formulas, CM represents the extrapolated center of mass (CoM) position accounting for velocity; pCoM and vCoM denote the position and velocity of the center of mass in the anterior-posterior and medial-lateral directions, respectively; g is the acceleration due to gravity; l is the vertical distance between the center of mass and the midpoint of the ankle joints; and B_max is the maximum boundary of the base of support in a given direction, which in this study is represented by the position of the center of pressure (CoP). In the sagittal plane, a positive Margin of Stability in the anterior and posterior directions (MoS_ap_) indicates that the velocity of the CoM is directed backwards and that the extrapolated center of mass lies anterior to the CoP. In the coronal plane, a positive Margin of Stability in the Medial and Lateral directions (MoS_ml_) indicates that the velocity of the CoM is directed outward and that the extrapolated center of mass lies medial to the CoP; negative values indicate the opposite. Therefore, a larger MoS value reflects a greater distance between the extrapolated center of mass and the CoP, corresponding to poorer dynamic stability (see Figure 3) (Hof et al., 2005).

#### 2.5.3 Data processing for work parameters

Work parameters were divided into two parts: the work performed by the CoM (reflecting the overall lower limb work) and the work performed by the three lower limb joints (hip, knee, and ankle joint work). All parameters were processed using Visual 3D software with a fourth-order Butterworth low-pass digital filter applied to kinematic and kinetic data, with cut-off frequencies set at 14 Hz and 50 Hz, respectively (Koblbauer et al., 2014; Winter, 2009). This study selected horizontal plane CoM work and sagittal plane work of the three lower limb joints during the stance phase for comparative analysis. To eliminate the effect of body weight on work results, all work parameters were normalized by dividing by the participant’s body weight. All parameters were further normalized by dividing by the corresponding external load magnitude to reflect the work performance per unit weight of load. The final calculated results represent work performance normalized by both body and load weights. The definitions and calculation procedures for the work parameters are as follows:

1. Calculation procedure for CoM work: First, calculate the CoM power per unit time. Then, the trapezoidal rule integrates the CoM power over time to obtain the total CoM work.

CoM power calculation formula (Winter, 2009):

Ci=Gi×Di

In the formulas, Ci, Gi, and Di represent the CoM power (unit: W), vGRF (unit: N), and center of gravity displacement (unit: m) of frame i, respectively.

CoM work calculation formula (Winter, 2009):

$$W\text{ positive}=\int_{start}^{end}PdtC>0;W\text{ negative}=\int_{start}^{end}PdtC<0$$

In the formulas, W positive is the positive work done, W negative is the negative work done, and the total work done = W positive + |W negative|.

2. Calculation procedure for the work of the three lower limb joints: First, calculate the joint power per unit time. Then, the trapezoidal rule integrates the joint power over time to obtain joint work.

Joint power calculation formula (using the hip joint as an example) (Zhang et al., 2024):

Pi=Mi×ωi

In the formulas, Pi, Mi, and ωi represent the hip joint power (unit: W), joint moment (unit: Nm), and joint angle velocity (unit: rad/s) at frame i, respectively.

Joint work calculation formula (using the hip joint as an example) (Winter, 2009):

$$W\text{ positive}=\int_{start}^{end}PdtPi>0;W\text{ negative}=\int_{start}^{end}PdtPi<0$$

In the formulas, W positive represents the positive work done by the hip joint, W negative represents the negative work done by the hip joint, and the total work done by the hip joint = W positive + |W negative|.

### 2.6 Statistical analysis

SPSS 25.0 software was used to perform tests for normality and homogeneity of variance. If the data met the assumptions of normal distribution and homogeneity of variance, two-way ANOVA (with Bonferroni *post hoc* tests) was conducted. If the data did not meet normality, non-parametric tests (Mann–Whitney U and Kruskal–Wallis tests) were applied. All data are presented as mean (standard deviation), and the level of statistical significance was set at α = 0.05. Based on previous studies (Cohen, 1973), partial eta squared (ŋ^2^) was used to report effect sizes, with 0.1–0.25 indicating a small effect, 0.25–0.4 a medium effect, and values greater than 0.4 a large effect.

## 3 Results

### 3.1 Results of dynamic stability

The ANOVA results for MoS_ap_ (Table 2) showed a significant main effect of external load magnitude [F (2, 8) = 19.991, P < 0.001, ŋ2 = 0.255] and a significant main effect of carrying technique [F (2, 8) = 9.819, P < 0.001, ŋ^2^ = 0.144]. However, the interaction between external load magnitude and carrying technique was not significant [F (4, 8) = 2.449, P = 0.051, ŋ^2^ = 0.077]. Post hoc analysis of external load magnitude indicated that MoS_ap_ during 30 kg load tasks was significantly greater than during 20 kg and 10 kg load tasks, and MoS_ap_ during 20 kg load tasks was significantly greater than during 10 kg load tasks. Post hoc analysis of carrying technique showed that MoS_ap_ under the hand-carry was significantly greater than under the shoulder and bosom-carry.

**TABLE 2:** Results of analysis of variance for dynamic stability (N = 126).

Parameters (m)	10 kg	20 kg	30 kg	P value	Shoulder-carry	Bosom-carry	Hand-carry	P value	Interaction P value
MoS_ap_	−1.08 (0.16)	−0.98 (0.14)	−0.89 (0.15)	<0.001	−1.02 (0.11)	−1.02 (0.12)	−0.90 (0.22)	<0.001	0.051
MoS_ml_	−0.10 (0.04)	−0.12 (0.05)	−0.10 (0.03)	0.074	−0.09 (0.03)	−0.10 (0.04)	−0.13 (0.05)	<0.001	0.025

**FIGURE 4:**
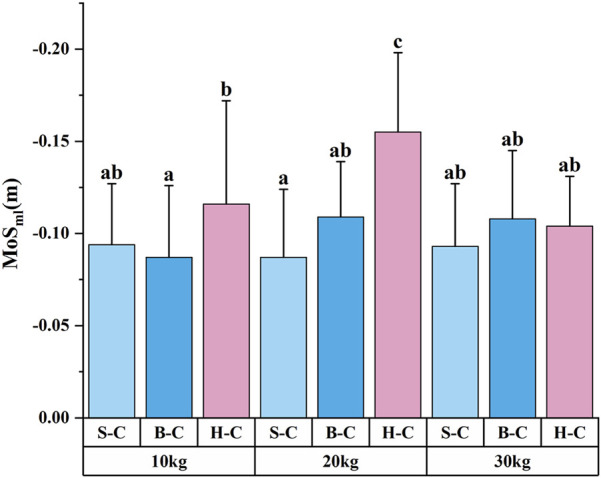
Results of multiple comparisons of the interaction effect of dynamic stability parameters (N = 126). The results presented include only the multiple comparisons of indicators showing significant interaction effects. In the figure, identical letter markings indicate no significant difference (P > 0.05), while the absence of identical letters indicates a significant difference (P < 0.05); S-C, B-C, and H-C refer to the Shoulder-carry, Bosom-carry, and Hand-carry, respectively.

The ANOVA results for MoS_ml_ (Table 2) revealed that the main effect of external load magnitude was not significant [F (2, 8) = 2.663, P = 0.074, ŋ^2^ = 0.044], while the main effect of carrying technique was significant [F (2, 8) = 8.643, P < 0.001, ŋ^2^ = 0.129]. Moreover, there was a significant interaction between external load magnitude and carrying technique [F (4, 8) = 2.902, P = 0.025, ŋ^2^ = 0.090]. Post hoc analysis of the carrying technique showed that MoS_ml_ under the shoulder and bosom-carry techniques were significantly higher than those under the hand-carry technique. Further *post hoc* analysis of the interaction effect (Figure 4) revealed that MoS_ml_ was significantly higher using the hand-carry technique during 20 kg load tasks than in other task conditions. MoS_ml_ was significantly higher under the hand-carry technique with 10 kg loads than the bosom-carry technique with 10 kg loads or the shoulder-carry technique with 20 kg loads.

### 3.2 Results of work performance parameters

**FIGURE 5:**
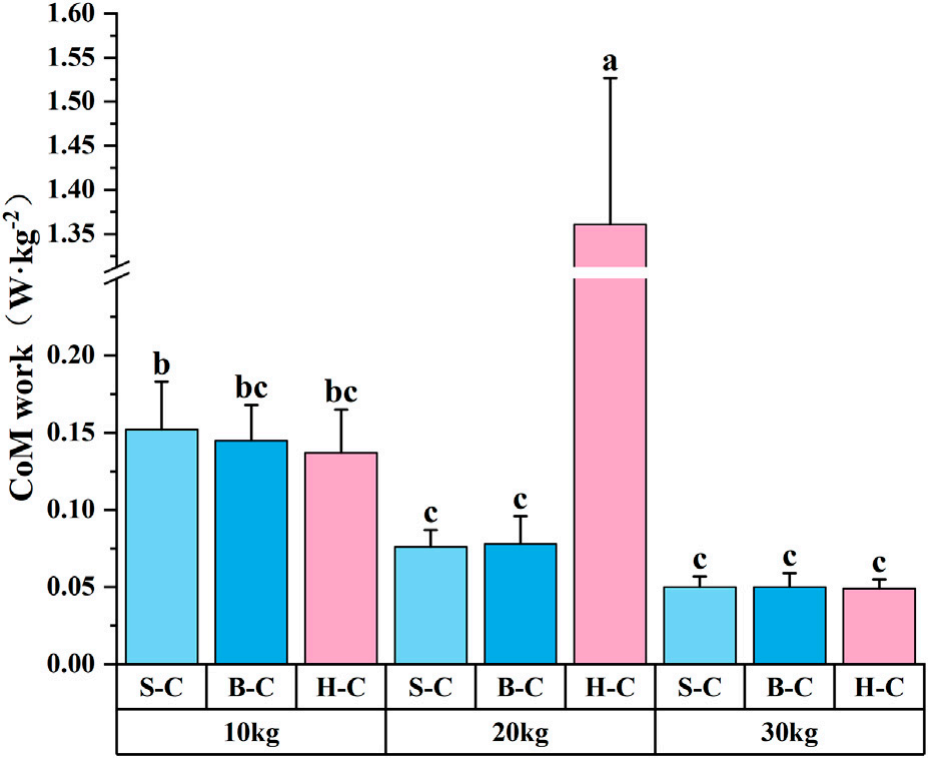
Results of multiple comparisons of the interaction effect of CoM work parameters (N = 126). The results presented include only the multiple comparisons of indicators showing significant interaction effects. In the figure, identical letter markings indicate no significant difference (P > 0.05), while the absence of identical letters indicates a significant difference (P < 0.05); S‐C, B‐C, and H‐C refer to the Shoulder‐carry, Bosom-carry, and Hand-carry, respectively.

The analysis of variance for CoM work revealed significant main effects of external load magnitude [F (2, 8) = 715.704, ŋ^2^ = 0.924] and carrying technique [F (2, 8) = 743.766, ŋ^2^ = 0.927], as well as a significant interaction between external load magnitude and carrying technique [F (4, 8) = 764.503, ŋ^2^ = 0.963]. Post hoc analysis for external load magnitude indicated that CoM work was significantly higher during 20 kg load tasks than during 30 and 10 kg load tasks. CoM work during 10 kg load tasks was significantly higher than during 30 kg load tasks. Post hoc analysis for carrying technique showed that the hand-carrying technique resulted in significantly higher CoM work than the shoulder and bosom-carrying techniques. Regarding the interaction effect (see Figure 5), the hand-carrying technique combined with a 20 kg load produced significantly higher CoM work than all other tasks. Additionally, performing the 10 kg load task with the shoulder-carrying technique resulted in significantly higher CoM work than performing 20 or 30 kg load tasks with the shoulder-carrying technique, 20 or 30 kg load tasks with the bosom-carrying technique, and 30 kg load tasks with the hand-carrying technique.

The analysis of variance for hip joint work (Table 3) revealed a significant main effect of external load magnitude [F (2, 8) = 52.978, P < 0.001, ŋ^2^ = 0.475] and a significant main effect of carrying technique [F (2, 8) = 3.290, P = 0.041, ŋ^2^ = 0.053]. In contrast, the interaction between external load magnitude and carrying technique was not significant [F (4, 8) = 1.543, P = 0.194, ŋ^2^ = 0.050]. Post hoc comparisons for external load magnitude showed that hip joint work was significantly higher in the 10 kg load condition compared to both the 20 and 30 kg load conditions, and higher in the 20 kg load condition than in the 30 kg load condition. Post hoc comparisons for carrying technique indicated that the hand-carrying technique resulted in significantly greater hip joint work than the bosom-carrying technique.

**TABLE 3:** Results of analysis of variance for work parameters (N = 126).

Parameters (W·kg^-2^)	10 kg	20 kg	30 kg	P value	Shoulder-carry	Bosom-carry	Hand-carry	P value	Interaction P value
CoM work	0.14 (0.03)	0.51 (0.62)	0.05 (0.01)	<0.001	0.09 (0.05)	0.09 (0.04)	0.52 (0.61)	<0.001	<0.001
Hip joint work	1,369.96 (616.14)	782.41 (234.20)	539.62 (151.04)	<0.001	871.27 (397.19)	806.39 (423.71)	1,014.33 (685.74)	0.041	0.194
Knee joint work	3,898.41 (15,597.77)	774.92 (256.15)	518.43 (158.96)	0.164	3,444.80 (15,672.97)	914.99 (489.61)	831.97 (485.39)	0.322	0.400
Ankle joint work	4,064.38 (14,042.28)	976.30 (200.32)	619.91 (128.14)	0.106	3,334.39 (14,163.96)	1,175.41 (518.69)	1,150.79 (697.32)	0.370	0.422

The results only present multiple comparison outcomes for indicators with significant interaction effects. In the figure, identical letter labels indicate no significant difference (P > 0.05), while the absence of identical letter labels indicates a significant difference (P < 0.05); S-C, B-C, and H-C refer to the Shoulder-carry, Bosom-carry, and Hand-carry, respectively.

The analysis of variance for knee joint work (Table 3) showed that the main effect of external load magnitude was not significant [F (2, 8) = 1.839, P = 0.164, ŋ^2^ = 0.030], the main effect of carrying technique was also not significant [F (2, 8) = 1.145, P = 0.322, ŋ^2^ = 0.019], and the interaction between external load magnitude and carrying technique was not significant either [F (4, 8) = 1.021, P = 0.400, ŋ^2^ = 0.034].

The analysis of variance for ankle joint work (Table 3) revealed that the main effect of external load magnitude was not significant [F (2, 8) = 2.291, P = 0.106, ŋ^2^ = 0.038], the main effect of carrying technique was not significant [F (2, 8) = 1.003, P = 0.370, ŋ^2^ = 0.017], and the interaction between external load magnitude and carrying technique was also not significant [F (4, 8) = 0.979, P = 0.422, ŋ^2^ = 0.032].

## 4 Discussion

### 4.1 Independent effects of external load magnitude and carrying technique on dynamic stability and work performance

In terms of dynamic stability, the results of this study indicate that external load magnitude had no significant effect on firefighters’ medial-lateral dynamic stability during fatigued rescue running tasks, but did have a significant impact on anterior-posterior stability. Specifically, smaller loads were associated with better dynamic stability in the anterior-posterior direction than moderate and large loads. From a biomechanical perspective, medial-lateral dynamic stability mainly relies on hip adduction or abduction control and pelvic lateral adjustment, both of which can be compensated for by trunk movements, making them more adaptable to load changes (Blackburn et al., 2003). In contrast, anterior-posterior stability depends more on step frequency control and propulsion ability. When firefighters carry heavier loads, increased inertia and mechanical burden affect their propulsion capability (Zhang et al., 2024), reducing anterior-posterior dynamic stability. Conversely, under small loads, the reduced inertia and mechanical stress are more conducive to efficient CoM adjustment (Winter, 2009). Furthermore, the study found that the carrying technique significantly influenced firefighters’ dynamic stability. Specifically, using the hand-carry technique resulted in poorer anterior-posterior stability but better medial-lateral stability. This is because bosom or shoulder-carrying techniques typically align the load’s gravitational line closer to the body’s central axis, thereby reducing anterior-posterior moment (Tang et al., 2025). However, compared to unilateral loading from the hand-carry technique, bosom and shoulder-carrying distribute the load symmetrically, limiting trunk-limb coordination and reducing dynamic coupling, enhancing medial-lateral stability in hand carrying (Winter, 2009). These findings suggest that task-specific technique selection must balance directional stability and load control strategy to optimize performance and reduce injury risk.

Regarding work performance, the present study revealed that firefighters exhibited the greatest CoM work when performing tasks with moderate loads, and the lowest under large loads. This pattern may be attributed to different biomechanical regulation strategies employed in response to varying load magnitudes. Previous studies have shown that firefighters must simultaneously maintain movement flexibility and counteract inertial forces when carrying moderate loads, necessitating sustained activation of core musculature to regulate and preserve dynamic balance. This leads to greater CoM oscillation and increased energy expenditure (Winter, 2009). Conversely, when performing tasks with large loads, the central nervous system activates protective compensatory mechanisms, such as enhancing core stiffness coupling and optimizing kinetic chain transmission efficiency. These strategies restrict CoM displacement in exchange for overall stability, thus reducing CoM work (Tang et al., 2025; Zhang et al., 2024). Notably, this study also found that among the three lower-limb joints, only the hip joint exhibited significant variation in work performance, with the highest under small loads and the lowest under large loads. This highlights the hip joint’s greater sensitivity to load changes, consistent with the “proximal joint dominance” principle in biomechanics (Winter, 2009). In contrast, the knee and ankle joints may primarily serve buffering and regulatory roles during task execution (Draper, 2012; Winter, 2009). As the load increases, the hip joint assumes more responsibility for pelvic stabilization and transmitting ground reaction forces (Han et al., 2010; Muscolino, 2022), leading to increased work output per unit load. Concerning carrying technique, results showed that firefighters using the hand-carry method had the highest CoM work, and again, only the hip joint showed significant variation in joint work, with hand-carrying generating more output than bosom. As previously discussed, the asymmetric load imposed by the hand-carry method forces the trunk to continuously resist lateral torque, triggering coronal plane balance adjustments (Winter, 2009). To maintain stability, the hip joint may need to execute combined abduction-abduction actions to counteract lateral momentum and coordinate multi-planar postural control. This multidimensional joint coordination increases systemic energy consumption and enhances lateral stability (Tang et al., 2025; Winter, 2009). In contrast, with its symmetrical load distribution, the bosom-carrying technique forms a more rigid kinetic chain that reduces trunk-limb dynamic coupling, thus lowering total work output, albeit at the cost of less effective lateral stability (Tang et al., 2025). These findings suggest that although the hand-carry technique enhances coronal plane stability, its asymmetric mechanical load may significantly increase biomechanical burden, particularly at the hip joint.

### 4.2 Interaction effects of external load magnitude and carrying technique on dynamic stability and work performance

In terms of dynamic stability, the present study found that the combination of carrying a moderate load using the hand-carry technique resulted in the poorest medial-lateral dynamic stability, potentially increasing the risk of gait deviation and lateral falls. As previously discussed, performing rescue tasks under moderate load requires firefighters to maintain movement flexibility and resistance to load inertia. However, the hand-carry technique imposes greater demands on the upper limb and shoulder girdle musculature (Cheung et al., 2021; Muscolino, 2022) while simultaneously restricting pelvic adjustments and gait symmetry (Hampton et al., 2011), thereby posing greater challenges to postural control. In contrast, the hug technique combined with a small load and the shoulder-carry technique combined with a moderate load showed superior medial-lateral dynamic stability. This is because the hug technique positions the load’s center of mass closer to the body’s axis, enhancing stability control (Tang et al., 2025), while the shoulder-carry method allows the load to align with the lateral torso closely, enhancing trunk involvement in postural regulation (Tang et al., 2025), thus stabilizing dynamic balance under moderate load conditions. Regarding work performance, the study revealed that combining the hand-carry technique with a moderate load resulted in the highest CoM work, significantly exceeding all other task types. This phenomenon may stem from the synergistic effects between load characteristics and technical demands. As previously mentioned, firefighters carrying a moderate load using the hand-carry technique operate within a challenging dynamic balance zone, necessitating continuous compromise between mobility and resistance to ground reaction forces (Winter, 2009). The addition of the hand-carry technique forces the trunk into compensatory lateral flexion in the coronal plane to counterbalance the load’s lateral moment. At the same time, the hip joint engages in more frequent and complex abduction-adduction motions to coordinate coronal plane stability (Tang et al., 2025). These multidirectional compensatory adjustments intensify oscillations of the CoM, significantly increasing energy dissipation (Winter, 2009). In contrast, under large load conditions, although the hand-carry method still imposes asymmetrical loading, the central nervous system tends to activate protective compensatory strategies—such as enhancing core muscle rigidity and transforming the kinetic chain into a quasisingle-degree-of-freedom system—thereby suppressing multidimensional compensation and reducing CoM work (Winter, 2009). These findings suggest that the combination of moderate load and hand-carry technique amplifies the biomechanical system’s dynamic instability and joint energy demands, indicating the need to optimize load distribution strategies or implement intervention training programs.

## 5 Conclusion

This study investigated the effects of external load magnitude and carrying technique on firefighters’ dynamic stability and work performance during fatigued rescue sprint tasks. The findings indicate that performing tasks under small load conditions enhances anterior-posterior dynamic stability compared to moderate and large loads. In contrast, moderate loads significantly increase CoM work, which may compromise energy efficiency. Although the hand-carry technique improves medial-lateral dynamic stability, it reduces anterior-posterior stability and increases energy expenditure. In contrast, the bosom and shoulder-carry techniques lower energy output but limit lateral dynamic regulation. Notably, combining the hand-carry technique with moderate loads should be avoided due to its adverse effects on stability and work efficiency. The shoulder-carry technique for moderate-to-large loads and the bosom technique for small loads enhance dynamic stability while reducing energy expenditure. Based on these findings, it is recommended that firefighters select carrying techniques according to load magnitude during field operations: prioritize the shoulder-carry technique for moderate and large loads, the bosom technique for small loads, and avoid using the hand-carry method for moderate loads. Additionally, firefighters are advised to emphasize hip joint functional training in their daily routines to enhance postural control, reduce injury risk, and improve task performance during rescue tasks.

## Data Availability

The original contributions presented in the study are included in the article/supplementary material, further inquiries can be directed to the corresponding authors.
